# Translation, cultural adaptation and validation of the Brazilian version of the Frontal Fibrosing Alopecia Quality of Life Index (FFA-QLI-BRA)^☆^

**DOI:** 10.1016/j.abd.2024.03.014

**Published:** 2024-11-08

**Authors:** Paula Rosa Coutinho Goulart Borges Mariottoni, Leonardo Spagnol Abraham, Leopoldo Duailibe Nogueira Santos, Daniel Fernandes Melo, Rodrigo Pirmez, Paulo Müller Ramos, Hélio Amante Miot

**Affiliations:** a Department of Dermatology, Trichosis Outpatient Clinic, Faculdade de Medicina de Botucatu, Universidade Estadual Paulista, Botucatu, SP, Brazil; b Trichosis Outpatient Clinic, Hospital Regional da Asa Norte, Brasília, DF, Brazil; c Department of Dermatology, Alopecia Outpatient Clinic, Santa Casa de São Paulo, SP, Brazil; d Department of Dermatology, Trichosis Outpatient Clinic, Universidade Estadual do Rio de Janeiro, Rio de Janeiro, RJ, Brazil; e Centro de Estudos do Cabelo, Santa Casa da Misericórdia do Rio de Janeiro, RJ, Brazil; f Department of Infectology, Dermatology, Imaging Diagnosis and Radiotherapy, Faculty of Medicine, Universidade Estadual Paulista, Botucatu, SP, Brazil

*Dear Editor,*

Described in 1994, frontal fibrosing alopecia (FFA) is a primary lymphocytic cicatricial alopecia primarily characterized by anterior hairline recession and bilateral eyebrow loss. FFA most frequently affects postmenopausal women (87%) and its incidence has been progressively increasing, making it currently the main cause of cicatricial alopecia treated by dermatologists.^1^, ^2^

Although it does not carry a risk of death or contagion, alopecias have a great impact on Quality of Life (QoL). This impact is evidenced by the higher prevalence of depressive symptoms and anxiety in patients with alopecia, especially among women.^3^, ^4^

Few studies have investigated the impact of QoL induced by FFA, but most patients recognize that their QoL is affected by it.^5^ Porriño-Bustamante et al. developed and validated a specific instrument to investigate the impact on QoL in women with FFA, the Frontal Fibrosing Alopecia Quality of Life Index (FFA-QLI).^6^ This self-administered instrument consists of 20 items with four alternatives, resulting in a score (one-dimensional) of the items ranging from 0 to 60 points.

A methodological study was carried out to adapt and validate the Brazilian version of the FFA-QLI. After authorization by the instrument authors, two dermatologists, fluent in English, translated it into Brazilian Portuguese, generating a consensus version, which was back-translated into English by a non-specialist and approved by the authors of the original questionnaire. The Brazilian version was named FFA-QLI-BRA. To obtain the cultural adaptation, ten women with FFA evaluated the instrument and were asked about the clarity of the questions, the language used, and its applicability. The version adapted to Brazilian Portuguese is available at https://doi.org/10.17632/75v2xxrb39.1.

For content validation, seven dermatologists with experience in trichology evaluated and scored each item (0 to 10) for relevance, with zero corresponding to ‘Not at all relevant’ and ten to ‘Very relevant’.

For the other validations, 99 women with FFA (diagnosed by a dermatologist) were investigated using an online questionnaire containing demographic data, FFA-QLI-BRA, and the Dermatology Quality of Life Index (DLQI-BRA), for concurrent validation. The study was conducted at the Dermatology Service of FMB-Unesp (Botucatu, SP, Brazil). A subgroup of eight participants completed the questionnaire again within a seven-day period, aiming to investigate its temporal stability. The instrument internal consistency was assessed using the McDonald-ω coefficient.

The demographic and QoL findings of the sampled patients are shown in Table 1. There was a predominance of women over 30 years of age, with higher levels of schooling. During the in-person application, the instrument was self-completed in less than ten minutes by all participants.

**Table 1:** Demographic and quality of life data of 99 women with frontal fibrosing alopecia.

Variables		Values
Age (years)	Mean (SD)	54 (11)
Level of schooling^a^	Elementary education	7 (7%)
	High School	17 (17%)
	Higher Education	75 (76%)
Marital status^a^	Married	73 (74%)
	Divorced	20(20%)
	Single	6 (6%)
DLQI-BRA	Median (Q1‒Q3)	3 (1‒7)
FFA-QLI-BRA	Mean (SDP)	24 (12)

a n (%), SD, Standard Deviation, Q1‒Q3, First and Third Quartiles.

The scores for each item of the FFA-QLI-BRA are shown in Fig. 1. Item F19 (regarding eyebrow makeup) showed a ceiling effect. The floor effect was observed in items 11, 14 and 20, related to leisure/sports activities, relationships with friends, and use of hair protheses.

**Fig. 1:**
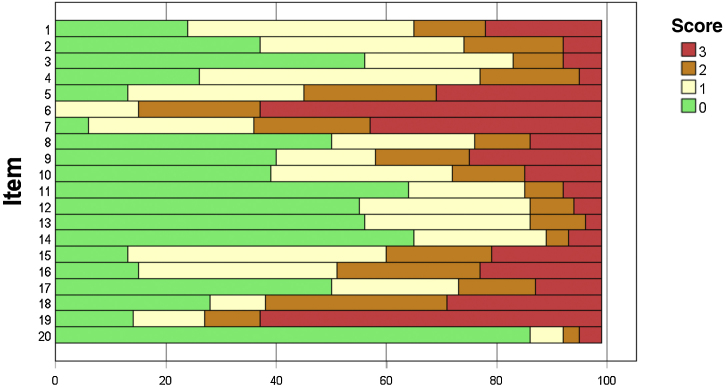
Distribution of item scores in the FFA-QLI-BRA (n = 99).

The content validity was ratified by an average > 8 from the experts for all items of the instrument.

The internal consistency (McDonald-ω) of the FFA-QLI-BRA was 0.92 (95% CI 0.90‒0.94) and of the DLQI was 0.89 (95% CI 0.86‒0.92). The correlation (Spearman's rho) between the FFA-QLI-BRA and DLQI scores was 0.77 (p < 0.001). The correlation of the FFA-QLI-BRA item scores among themselves and between the items and the total score (Fig. 2) indicates lower coefficients related to items E4, F19 and F20.

**Fig. 2:**
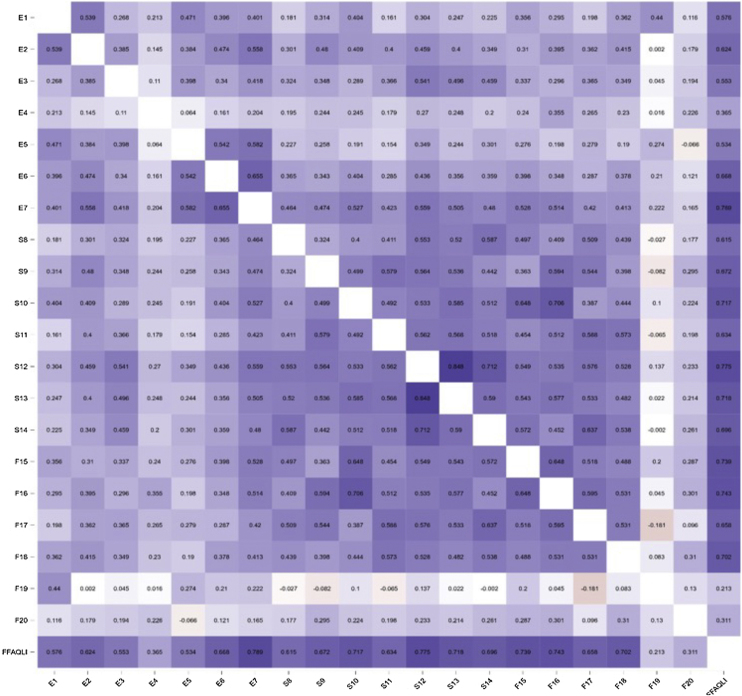
Correlation heatmap (Spearman's rho) between FFA-QLI-BRA items and the total score (n = 99).

The exploratory factor analysis, using the principal axis factoring method, indicated that 48.9% of the variance of the latent variable was explained by the unidimensional factor. The KMO coefficient for the matrix was 0.86 and Bartlett's sphericity test resulted in p < 0.001, indicating sample adequacy. All items showed factor loading ≥ 0.34, except F19, which resulted in 0.16.

The network analysis using the EBICglasso method (Fig. 3) shows centrality for the FFA-QLI-BRA items, especially S9, S13, and F17. Items from the DLQI and E3-6, F19, and F20 were located peripherally in the construct representation.

**Fig. 3:**
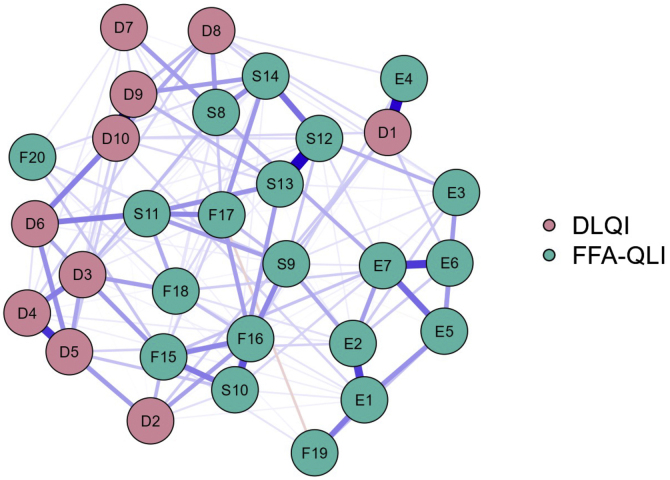
Network diagram between items of the DLQI-BRA and FFA-QLI-BRA (n = 99).

The exclusion of items E4, F19, and F20 increases the internal consistency to 0.93, and the variance of the explained latent variable to 57.9%.

In the temporal stability assessment, the mean (standard deviation) of the test scores was 16,^9^ and in the retests, it was 13,^9^ resulting in an intraclass correlation coefficient (agreement) of 0.80.

The psychometric analysis of the FFA-QLI-BRA showed that the instrument shows internal consistency, content validity, and temporal stability, validating its use in Brazil. This allows a more precise quantification of these patients demand perception, improving their care.

In this sample, the impact assessed by the DLQI was mild, similar to that described in the literature.^7^ However, generic questionnaires do not detect all the aspects affected in patients lives, leading to a preference for specific instruments, when available.^8^ The greater internal consistency and the position of the FFA-QLI-BRA items in the center of the network indicate a superior psychometric performance compared to the DLQI for the assessment of QoL in FFA.^9^

Certain items of the FFA-QLI-BRA showed psychometric behavior that was less correlated with the other items and should be carefully evaluated during the use of the instrument. Item F19 (eyebrow makeup) may fail in women without eyebrow involvement or may require daily care for those affected by eyebrow loss. Item E4 (pruritus/pain) may not be present, or may be mitigated with treatment, and does not seem to be an issue that compromises QoL. Item 20 can only be evaluated in cases that require hair prosthesis, being inconsistent in samples consisting of patients with mild disease.

In this study, the mean FFA-QLI-BRA scores indicated a moderate impact on QoL, while the original study in Spain indicated a mild impact.^6^ Further research should explore the correlation between QoL scores and clinical variables, cultural aspects, and affection diseases scores, since Latin American populations may have greater affection sensitivity to hair loss. A Brazilian study showed that womens fear of losing all their hair is similar to that of contracting serious diseases, such as heart disease.^10^

This study has limitations related to sampling women undergoing treatment and with a higher level of schooling than the general Brazilian population, which should alert to the need for a good understanding of the instrument when applied to women with a lower level of schooling. However, these elements did not prevent the instrument analysis and validation process.

In conclusion, the Brazilian version of the FFA-QLI was adapted and proved to be valid and consistent, establishing itself as an useful tool in clinical practice, as well as in studies involving patients with FFA.

## Financial support

None declared.

## Authors' contributions

Paula Rosa Coutinho Goulart Borges Mariottoni: Design and planning of the study; collection of data; drafting and editing of the manuscript; critical review of the literature; critical review of the manuscript; approval of the final version of the manuscript.

Leonardo Spagnol Abraham: Critical review of the literature; critical review of the manuscript; approval of the final version of the manuscript.

Leopoldo Duailibe Nogueira Santos: Critical review of the literature; critical review of the manuscript; approval of the final version of the manuscript.

Daniel Fernandes Melo: Critical review of the literature; critical review of the manuscript; approval of the final version of the manuscript.

Rodrigo Pirmez: Critical review of the literature; critical review of the manuscript; approval of the final version of the manuscript

Paulo Müller Ramos: Design and planning of the study; drafting and editing of the manuscript; critical review of the literature; critical review of the manuscript; approval of the final version of the manuscript.

Hélio Amante Miot: Design and planning of the study; analysis and interpretation of data; statistical analysis; drafting and editing of the manuscript; critical review of the literature; critical review of the manuscript; approval of the final version of the manuscript.

## Conflicts of interest

None declared.
